# Comparative *in vitro* antibacterial activity of herbal extracts from *Piper betle* L., *Bauhinia scandens* L. and *Chromolaena odorata* against *Staphylococcus aureus*, *Escherichia coli* and *Pseudomonas aeruginosa*

**DOI:** 10.7717/peerj.21134

**Published:** 2026-04-20

**Authors:** Sokom Kong, Jaturong Wongsanit, Praphaphan Krajanglikit, Nayika Pinniam, Prakorn Jara, Siriluk Jala, Suporn Thongyuan, Phitsanu Tulayakul

**Affiliations:** 1 Faculty of Veterinary Medicine, Kasetsart University, Bangkok, Thailand; 2 Department of Large Animal and Wildlife Medicine, Faculty of Veterinary Medicine, Kamphaeng Saen Campus, Kasetsart University, Nakhon Pathom, Thailand; 3 Department of Veterinary Public Health, Faculty of Veterinary Medicine, Kamphaeng Saen Campus, Kasetsart University, Nakhon Pathom, Thailand; 4 Kamphaeng Saen Veterinary Diagnostic Center, Faculty of Veterinary Medicine, Kamphaeng Saen Campus, Kasetsart University, Nakhon Pathom, Thailand

**Keywords:** Antibacterial activity, Herbal extracts, *Bauhinia scandens* L., *Piper betle* L., *Chromolaena odarata*, *Staphylococcus aureus*, *Escherichia coli*, *Pseudomonas aeruginosa*

## Abstract

**Background:**

Antimicrobial resistance remains a major global challenge and has increased interest in plant-derived compounds as alternative or complementary therapeutic agents. This study evaluated the *in vitro* antibacterial activity of ethanol-extracted *Piper betle* L. or betel leaf (EPB), *Bauhinia scandens* L. stem (EBS), and *Chromolaena odorata* leaf (ECO) against common mastitis-associated pathogens.

**Methods:**

The three herbal samples were prepared through ethanol extraction followed by freeze-drying. Antibacterial activity was assessed using disc diffusion assays at two extract concentrations. Minimum inhibitory concentrations (MIC) and minimum bactericidal concentrations (MBC) were determined through broth microdilution. Statistical analyses were performed to compare the inhibitory and bactericidal performance of the extracts.

**Results:**

The extracts showed distinct antibacterial profiles. EPB and EBS produced the strongest inhibition zones against *Staphylococcus aureus (S. aureus)*, while ECO displayed weaker activity. In agreement with diffusion results, EPB and EBS had the lowest MIC and MBC values for *S. aureus*, and both showed bactericidal MBC/MIC ratios. Activity against *Escherichia coli (E. coli)* was limited; measurable inhibition was observed only for EPB, and all extracts required substantially higher MIC and MBC concentrations compared with *S. aureus*. None of the extracts demonstrated meaningful activity against *Pseudomonas aeruginosa (P. aeruginosa)*. Across assays, inhibition zone size showed a negative correlation with MIC values, indicating consistency between diffusion and broth-based methods.

**Conclusions:**

EPB and EBS demonstrated promising antibacterial activity against *S. aureus*, suggesting potential use as plant-based candidates for controlling Gram-positive mastitis pathogens. Their limited effects on Gram-negative bacteria (*E. coli* and *P. aeruginosa*) indicate they may be best suited for targeted rather than broad-spectrum applications. Further phytochemical characterization and *in vivo* studies are warranted to evaluate their therapeutic potential in livestock health management.

## Introduction

Antimicrobial resistance (AMR) has been increasingly reported among pathogenic bacteria worldwide. Among them, *Staphylococcus aureus* (*S. aureus*), *Escherichia coli* (*E. coli*) and *Pseudomonas aeruginosa* (*P. aeruginosa*), have emerged as significant global health challenges. It is affecting both humans and animals in many aspects. These pathogens are capable of causing severe infections and have shown increasing resistance to conventional antibiotics (Ahmed et al., 2024; Salam et al., 2023), prompting the need to consider alternative therapeutic strategies. In addition, the World Health Organization has identified AMR as one of the leading global health threats. This is emphasizing the pressing need for innovative approaches that can be used to mitigate this growing crisis as mentioned, including the exploration of plant-derived phytotherapeutics agent (World Health Organization, 2024).

Although synthetic antibiotics were initially highly effective, their clinical utility has declined due to the rapid emergence of bacterial resistance. Resistance mechanisms such as efflux pump activity, enzymatic degradation, and structural modification of antibiotic targets have substantially reduced the efficacy of many drug classes including *β*-lactams, fluoroquinolones and peptide antibiotics (Hooper & Jacoby, 2016; Kim & Jeon, 2016). As reported by Mukhopadhyay, Wade & Puopolo (2019), in addition to resistance, antibiotics are associated with toxicity, allergic reactions, and adverse side effects in both humans and animals, which restricts their safe therapeutic application. Together, these limitations highlight a clear need for safer, effective, and sustainable antibacterial alternatives.

Medicinal plants have gained attention as promising alternatives due to their diverse bioactive compounds and long history of use in traditional medicine for treating infections. Among these, *Piper betle* L. (*P. betle* L.) Or betel leaf, *Bauhinia scandens* L. (*B. scandens* L.) stem and *Chromolaena odorata* (*C. odorata*) leaf are widely used in Southeast Asia for their antimicrobial, anti-inflammatory, and wound-healing properties (Islam et al., 2022; Madhumita, Guha & Nag, 2019; Olawale, Olofinsan & Iwaloye, 2022). *P. betel* L. is rich in phenolic compounds such as chavicol and eugenol, which exhibit strong antibacterial activity (Singh et al., 2023). *B. scandens* L., traditionally used to treat inflammation and infections, contains flavonoids and saponins with potential antimicrobial effects (Islam et al., 2022). *C. odorata*, commonly known as Siam weed, has been documented for its wound healing and antibacterial activities, attributed mainly to the presence of flavonoids and essential oils (Olawale, Olofinsan & Iwaloye, 2022).

Bacitracin, enrofloxacin, and cefazolin are commonly used antibiotics in both human and veterinary medicine and were included in this study as reference compounds. Enrofloxacin, a fluoroquinolone that targets bacterial DNA replication (Hooper & Jacoby, 2016; Wolfson & Hooper, 1985), and cefazolin, a first-generation cephalosporin that inhibits cell wall synthesis (Mukhopadhyay, Wade & Puopolo, 2019), included to provide standardized benchmarks for evaluating the antibacterial performance of the tested herbal extracts.

Despite the reported antimicrobial properties of these plants, direct comparative *in vitro* studies evaluating their antibacterial efficacy against mastitis-associated bacterial reference strains under identical experimental conditions and using standardized susceptibility parameters remain limited.

Therefore, this study provides a novel, direct comparative evaluation of the *in vitro* antibacterial activity of ethanol extracts from *P. betle* L. leaf (EPB), *B. scandens* L. stem (EBS), and *C. odorata* leaf (ECO) against reference strains of *S. aureus*, *E. coli*, and *P. aeruginosa*.

We hypothesized that EPB and EBS would exhibit stronger antibacterial activity against the Gram-positive pathogen *S. aureus* than against Gram-negative bacteria. Antibacterial efficacy was assessed using disc diffusion assays, minimum inhibitory concentration (MIC), and minimum bactericidal concentration (MBC) determinations.

## Materials & Methods

### Bacterial strains

Reference isolates of *S. aureus* (DMST 8840), *E. coli* (DMST 4212), and *P. aeruginosa* (DMST 4739) were obtained from the Department of Medical Sciences Thailand (DMST) culture collection and maintained in the bacteriology laboratory of the Faculty of Veterinary Medicine, Kasetsart University, Bangkok, Thailand. All the strains were confirmed using standard microbiological and biochemical methods before experimentation.

### Preparation of the herbal extracts

Fresh samples of *P. betle* L. leaves, *B. scandens L.* stems, and *C. odorata* leaves were collected during the dry season (January to March 2024) from cultivated and wild-growing areas in Thailand, and washed thoroughly with distilled water.

After shade-drying at room temperature, the plant materials were cut into small pieces (for leaves) or ground directly (for stems), then oven-dried at 50 °C for 12–24 h (KG, Model 700; Memmert GmbH + Co., Schwabach, Germany). The dried materials were powdered using a sterile electric blender, sieved at 125 µm, and stored in a desiccator until use.

For extraction, 10 g of EPB, EBS, or ECO powder was mixed with 100 mL of 99% ethanol (1:10 ratio, 0.1 g/mL) or with 50 mL of 99% ethanol (1:5 ratio, 0.2 g/mL). Ethanol was selected as the extraction solvent because it efficiently solubilizes a broad range of bioactive phytochemicals, including phenolics, flavonoids, alkaloids, and terpenoids, which are commonly associated with antimicrobial activity. In addition, ethanol is relatively low in toxicity, compatible with microbiological assays, and can be readily removed under reduced pressure (Kozhantayeva et al., 2024; Lee et al., 2024).

Each extraction was shaken in an incubator shaker at 200 rpm for 24 h at room temperature, filtered through a 0.45 µm cellulose acetate filter (Sartorius), and concentrated using a rotary evaporator at 50 °C. The extracts were then freeze-dried to obtain stable dry crude extracts. Freeze-drying was chosen to remove residual solvent and moisture under low-temperature conditions, thereby minimizing thermal degradation of heat-sensitive phytochemicals and improving extract stability. This process also enabled accurate reconstitution and concentration standardization for subsequent antimicrobial assays (MIC and MBC). The dried extracts were stored at 4 °C until analysis (Babaei Rad et al., 2025).

Concentrations expressed in g/mL refer to the weight-to-volume (w/v) ratio of crude plant material during ethanol extraction for disc diffusion assays, whereas MIC and MBC values are reported in µg/mL to reflect the final concentrations of dried extracts used in broth microdilution testing.

### Preparation of the antibiotic standards

Bacitracin (PHR1590-1G, CAS-No: 1405-87-4; Merck, Shanghai, China), octyl gallate (48700-50G-F, CAS-No: 1034-01-1; Merck), enrofloxacin, and cefazolin were used as reference antibiotics. Stock solutions were prepared according to the manufacturer’s instructions and stored at −20 °C until required.

### Disk diffusion method

The antimicrobial activities of the herbal extracts and standard antibiotics against *S. aureus*, *E. coli*, and *P. aeruginosa* were evaluated using the disk diffusion method. Blood agar plates were prepared and sterilized before inoculation with bacterial suspensions adjusted to a 0.5 McFarland standard. Sterile cotton swabs were used to uniformly streak the bacterial cultures across the agar surface. Paper disks impregnated with herbal extracts of *Piper betle* L. (betel leaf) (PBL), *Bauhinia scandens* L. (BSL), and *Chromolaena odorata* (CO) (EPB, EBS, ECO) and standard antibiotics (bacitracin and octyl gallate) were placed on plates using sterile forceps, ensuring appropriate spacing between disks. Solvent-only disks were included as negative controls. The plates were incubated at 37 °C for 16–18 h, and inhibition zones were measured in millimeters. All tests were performed in triplicate. Results were interpreted following the Clinical and Laboratory Standards Institute (CLSI) guidelines (CLSI, 2024), and control strains were used to validate assay quality.

### Determination of the minimum inhibitory concentration (MIC)

The MIC values of the herbal extracts and antibiotics were determined using the broth microdilution method following the CLSI guidelines (CLSI, 2012; CLSI, 2024). Based on disk diffusion results, MIC testing was performed for *S. aureus* and *E. coli* using the 0.2 g/mL or 1:5 (in ratio) freeze-dried ethanolic herbal extracts.

The bacterial suspensions were prepared in Mueller-Hinton broth (MHB) and adjusted to a turbidity equivalent to 0.5 McFarland standard (approximately 1  × 10^8^ CFU/mL). The suspensions were further diluted in MHB to obtain a final inoculum of 5  × 10^5^ CFU/mL per well, in accordance with CLSI recommendations. Twofold serial dilutions of the extracts and antibiotics were prepared to obtain final concentrations expressed in µg/mL. Each well was inoculated with 100 µL of the bacterial suspension and 100 µL of the test compound, resulting in a final bacterial concentration of 5 × 10^5^ CFU/mL per well. The plates were incubated at 37 °C for 18 to 24 h. The MIC was defined as the lowest concentration of the extract or antibiotic that visibly inhibited bacterial growth.

### Determination of the minimum bactericidal concentration (MBC)

To determine the MBC 10 µL aliquots were withdrawn from wells showing no visible growth after MIC determination, and streaked onto blood agar (for Gram-positive bacteria) or MacConkey agar (for Gram-negative bacteria). The plates were incubated at 37 °C for 24 h.

The MBC was defined as the lowest concentration of the herbal extract or antibiotic at which no visible colony growth was observed on the agar surface, indicating complete bacterial killing (≥99.9% reduction in the initial inoculum). All the tests were performed in triplicate. This procedure followed CLSI guideline M26-A: Methods for Determining Bactericidal Activity of Antimicrobial Agents.

### Quality control

Sterility controls (broth without bacteria) and growth controls (broth with bacteria but without extract or antibiotic) were included in each assay. All the experiments were performed in triplicate to ensure reproducibility.

### Data analysis

Inhibition zones diameters obtained from disc diffusion assays were expressed as mean ± standard deviation (SD) from three independent triplicates. Results were summarized as mean ± SD to represent central tendency and inter-replicate variability, as biological assays such as disc diffusion are subject to experimental variation arising from inoculum density, agar diffusion dynamics, and measurement precision. Reporting SD allows assessment of data dispersion and reproducibility across independent experiments.

Independent *t-tests* were used to compare inhibition zones between two concentrations of the same herbal extract when assumptions of normality and homogeneity of variance were satisfied. Comparisons among multiple herbal extracts tested against the same bacterial species were performed using one-way analysis of variance (ANOVA) followed by Tukey’s *post-hoc* test for pairwise comparisons.

For datasets characterized by zero-inflated values or non-normal distributions (for instance, absence of measurable inhibition against *P. aeruginosa*), the Kruskal–Wallis rank-sum test was applied. This non-parametric method was selected because it does not assume normally distributed residuals and is appropriate for small sample sizes and skewed data containing multiple zero values.

MIC and MBC values were reported descriptively as mean ± SD. Inferential statistical testing was not performed for MIC and MBC because these values were derived from discrete twofold serial dilution assays, which generate ordinal concentration endpoints and were based on limited replicate numbers.

Pearson’s correlation analysis was performed to assess the relationship between inhibition zone diameters at 0.2 g/mL and corresponding MIC values for each extract–bacterium pair. Pearson’s method was selected because both variables were treated as continuous quantitative measurements and the analysis aimed to evaluate the strength and direction of a linear association between disc diffusion activity and broth microdilution potency. The test is appropriate when assessing linear relationships under approximate normality and homoscedasticity assumptions, and it allows quantification of correlation magnitude through the correlation coefficient (*r*). Statistical significance was set at *p* < 0.05.

All analyses were performed using R (version 4.4.2; R Core Team, 2024), which was selected due to its validated statistical libraries, reproducibility, and transparency in analytical workflows. Additional data processing and visualization were performed using RStudio, which provides an integrated environment for script management, graphical output, and reproducible research documentation.

### Writing

The manuscript was written primarily by the authors. The first author prepared the original draft, and all co-authors critically reviewed and revised the text. The manuscript was subsequently edited by a native English language reviewer prior to submission.

Limited use of ChatGPT (OpenAI, 2025) was made solely for minor language editing, including grammar correction and clarification of wording in selected sentences. The tool was used in a restricted manner and did not contribute to drafting scientific content, structuring the manuscript, or developing arguments.

No AI tools were used for data generation, analysis, interpretation, visualization, experimental design, or statistical evaluation. The AI tool did not influence the scientific conclusions of the study. All scientific content, interpretations, and conclusions are entirely the responsibility of the authors.

## Results

### Herbal inhibition

The antibacterial activity of the three herbal extracts such as EPB, EBS and ECO, was evaluated against *S. aureus*, *E. coli*, and *P. aeruginosa* using disc diffusion assays at two concentrations (0.1 and 0.2 g/mL). Overall, the extracts demonstrated variable inhibitory effects across bacterial species, with the strongest and most consistent activity observed against *S. aureus*. Both EPB and EBS produced clear inhibition zones at both concentrations, whereas ECO showed weaker but meanable activity.

In contrast, EPB was the only extract to show measurable inhibition against *E. coli*, while EBS and ECO produced no detectable zones at either concentration. at either concentration.

**Figure 1:**
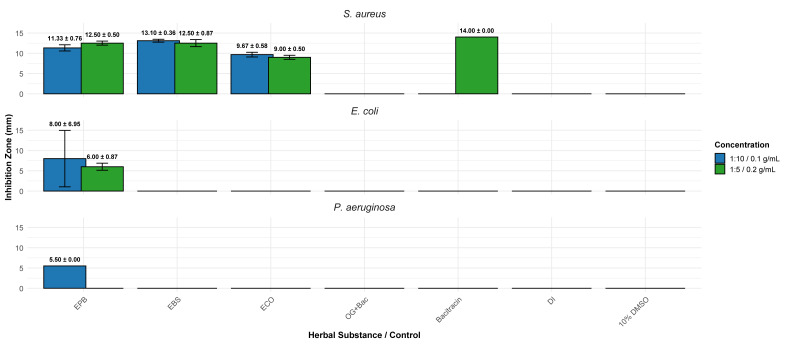
Inhibition zone diameters for *S. aureus*, *E. coli*, and *P. aeruginosa* treated with EPB, EBS, ECO, and the controls. Bars represent mean inhibition zone diameters (mm ± SD) from three independent experiments (*n* = 3) at two concentrations: 0.2 g/mL (1:5, w/v) and 0.1 g/mL (1:10, w/v). SD is standard deviation. EPB, ethanol extract of *Piper betle* leaf; EBS, ethanol extract of *Bauhinia scandens* L. stem; ECO, ethanol extract of *Chromolaena odorata* leaf. Bac, bacitracin; OG, octyl gallate; DI, distilled water; 10% DMSO = 10% dimethyl sulfoxide.

**Figure 2:**
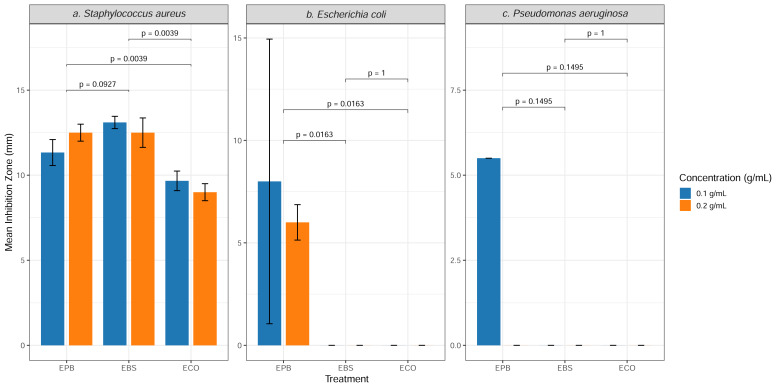
(A–C) Antibacterial activity of herbal extracts against mastitis-associated bacteria. Antibacterial activity of EPB, EBS, and ECO against mastitis-associated bacteria (*Staphylococcus aureus*, *Escherichia coli*, and *Pseudomonas aeruginosa*). Bars represent mean inhibition zone diameters (mm ± SD) at 0.1 g/mL and 0.2 g/mL from three independent experiments (*n* = 3), and individual points indicate biological replicates.Pairwise statistical comparisons between extracts within each bacterial species at the same concentration were performed using the Kruskal–Wallis test followed by *post hoc* analysis. Brackets indicate comparisons, with corresponding exact *p*-values shown; *p* < 0.05 was considered statistically significant. EPB, ethanol extract of *Piper betle* leaf; EBS, ethanol extract of *Bauhinia scandens* L. stem; ECO, ethanol extract of *Chromolaena odorata* leaf. Concentrations expressed in g/mL represent the weight-to-volume (w/v) ratio of crude extract prepared for disc diffusion assays.

Activity against *P. aeruginosa* was minimal; EPB showed weak inhibition at 0.1 g/mL but no effect at 0.2 g/mL, and neither EBS nor ECO generated inhibition at any concentration. Controls, including 10% DMSO, DI water and OG+Bacitracin, showed no activity, while Bacitracin inhibited only *S. aureus*. These findings indicate that EPB possesses the broadest antibacterial spectrum among the three extracts, whereas EBS and ECO are primarily active against Gram-positive *S. aureus* and exhibit limited or no activity against Gram-negative bacteria. These trends are summarized in Fig. 1 and supported by the extract-specific inhibition data provided in Fig. 2.

### Herbal inhibition of *S. aureus*

As shown in Fig. 1, all three herbal extracts inhibited *S. aureus*, with EBS and EPB producing the largest inhibition zones. From the experiment found that, at 0.1 g/mL, EBS produced the strongest inhibition (13.10 ± 0.36 mm), followed by EPB (11.33 ± 0.76 mm) and ECO (9.67 ± 0.58 mm). In addition, at 0.2 g/mL concentration, EPB showed a modest inhibition zone (12.50 ± 0.50 mm; *p* = 0.0178), while EBS and ECO exhibited slight reductions in zone diameter, both statistically significant (*p* = 0.001).

These quantitative results are presented in Table 1, with corresponding visual comparisons shown in Fig. 2, where pairwise statistical analysis demonstrated that EPB and EBS produced significantly greater inhibition than ECO (*p* = 0.0039). These results confirm superior activity against *S. aureus*.

**Table 1:** Comparison of inhibition zone diameters for EPB, EBS, and ECO at 0.1 g/mL and 0.2 g/mL against *S. aureus*. Inhibition zone diameters of EPB, EBS, and ECO against Staphylococcus aureus at 0.1 g/mL and 0.2 g/mL. Values represent mean ± standard deviation (mm) from three independent experiments (*n* = 3). *P*-values indicate comparisons between 0.1 and 0.2 g/mL within each extract using the Kruskal–Wallis test. A *p*-value < 0.05 was considered statistically significant.

Herb	Concentration (g/mL)	Mean (mm)	SD (mm)	*n*	*p*-value (0.1 *vs.* 0.2 g/mL)	Statistical significance
EPB	0.1	11.33	0.76	3	0.0178	*p* < 0.05
	0.2	12.50	0.50	3		
EBS	0.1	13.10	0.36	3	0.001	*p* < 0.05
	0.2	12.50	0.87	3		
ECO	0.1	9.67	0.58	3	0.001	*p* < 0.05
	0.2	9.00	0.50	3		

**Notes.**

EPB ethanol extract of *Piper betle* leaf EBS ethanol extract of *Bauhinia scandens* L. stem ECO ethanol extract of *Chromolaena odorata* leaf

The 0.2 g/mL concentration corresponds to a 1:5 (w/v) extraction ratio (1 g plant material in 5 mL ethanol), and 0.1 g/mL corresponds to a 1:10 (w/v) extraction ratio.

### Herbal inhibition of *E. coli*

The extracts displayed limited inhibition of *E. coli*, with measurable activity observed only for EPB. EPB produced variable inhibition at 0.1 g/mL (8.00 ± 6.95 mm) and more consistent inhibition at 0.2 g/mL (6.00 ± 0.87 mm), though this difference was not significant (*p* = 0.581).

The large standard deviation observed at 0.1 g/mL reflects high inter-replicate variability near the detection threshold, where inhibition was inconsistently observed across replicates. This variability likely reflects marginal antibacterial activity of EPB against *E. coli* at this concentration, rather than experimental error. EBS and ECO showed no measurable inhibitory effect at either concentration. These outcomes are summarized in Table 2 and visually supported by Fig. 2, where EBS and ECO remain at zero across all replicates. Statistical comparisons confirmed that EPB exhibited greater activity than with EBS and ECO against *E. coli*.

**Table 2:** Comparison of inhibition zone diameters for EPB, EBS, and ECO at 0.1 g/mL and 0.2 g/mL against *E. coli*.

Herb	Concentration (g/mL)	Mean (mm)	SD (mm)	*n*	*p*-value (0.1 *vs.* 0.2 g/mL)	Statistical significance
EPB	0.1	8.00	6.95	3	0.581	ns (not significant)
	0.2	6.00	0.87	3		
EBS	0.1	0	0	3	N/A	No inhibition
	0.2	0	0	3		
ECO	0.1	0	0	3	N/A	No inhibition
	0.2	0	0	3		

**Notes.**

EPB Extracts of *P. betel* leaf EBS Extracts of *Bauhinia scandens* L*.* stem ECO Extracts of *Chromolaena odorata* leaf, concentration of 0.2 g/mL = 1 gram of herbal powder + 5 mL of ethanol (1:5 ratio), concentration of 0.1 g/mL = 1 gram of herbal powder + 10 mL of ethanol (1:10 ratio) ns not significant (*p* > 0.05) N/A not applicable (cannot calculate *p*-value because all measurements are zero) n number of triplicates

### Herbal inhibition of *P. aeruginosa*

Only EPB demonstrated weak activity against *P. aeruginosa*, producing small inhibition zones (5.50 mm) at 0.1 g/mL, while no inhibition was detected at 0.2 g/mL. Moreover, neither EBS nor ECO produced any measurable zones at either concentration. The absence of measurable inhibition by EBS and ECO is consistent with the intrinsic resistance profile of *P. aeruginosa*, which possesses low outer membrane permeability and efficient efflux systems that restrict the activity of many phytochemicals. Besides the data displayed in Table 3, the inhibition pattern is clearly depicted in Fig. 1 (mean ± SD bar plots) and the raw-distribution analysis provided in Fig. 2. Together, these results indicate that all three herbal extracts possess limited or no activity against this species, consistent with the intrinsic resistance characteristics of *P. aeruginosa*. Based on the statistical analysis illustrated in Fig. 2, there was no difference of efficacy among the three herbs.

**Table 3:** Comparison of inhibition zone diameters for EPB, EBS, and ECO at 0.1 g/mL and 0.2 g/mL against *P. aeruginosa*. Inhibition zone diameters of EPB, EBS, and ECO against *Pseudomonas aeruginosa* at 0.1 g/mL and 0.2 g/mL. Values represent mean ± standard deviation (mm) from three independent experiments (*n* = 3). *P*-values indicate comparisons between 0.1 and 0.2 g/mL within each extract using the Kruskal–Wallis test. A *p*-value < 0.05 was considered statistically significant. “0.00” indicates no measurable inhibition under the tested conditions. The absence of activity for EBS and ECO likely reflects the intrinsic resistance mechanisms of *P. aeruginosa*, including reduced outer membrane permeability and active efflux systems, which limit susceptibility to many plant-derived compounds.

Herb	Concentration (g/mL)	Mean (mm)	SD (mm)	*n*	*p*-value (0.1 *vs.* 0.2 g/mL)	Statistical significance
EPB	0.1	5.50	0.00	3	0.046	*p* < 0.05
	0.2	0.00	0.00	3		
EBS	0.1	0.00	0.00	3	N/A	ns (not significant)
	0.2	0.00	0.00	3		
ECO	0.1	0.00	0.00	3	N/A	ns (not significant)
	0.2	0.00	0.00	3		

**Notes.**

EPB ethanol extract of *Piper betle* leaf EBS ethanol extract of *Bauhinia scandens* L. stem ECO ethanol extract of *Chromolaena odorata* leaf N/A not applicable (*p*-value cannot be calculated when all observations are zero)

The 0.2 g/mL concentration corresponds to a 1:5 (w/v) extraction ratio, and 0.1 g/mL corresponds to a 1:10 (w/v) extraction ratio.

### Minimum inhibitory concentration (MIC)

**Figure 3:**
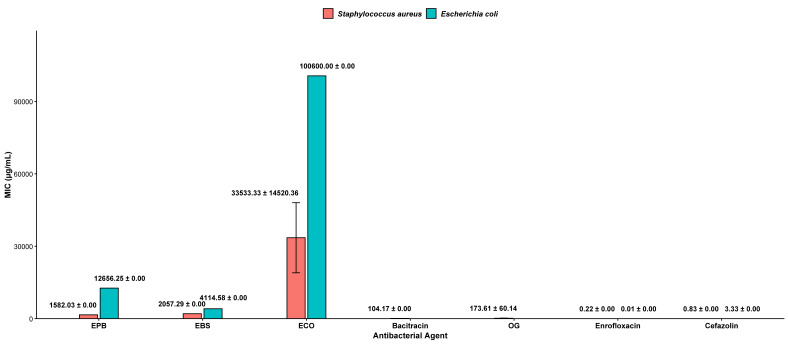
Minimum inhibitory concentration (MIC) test using EPB, EBS and ECO for *S. aureus* and *E. coli*. Bars represent mean MIC values (µg/mL ± standard deviation [SD]) determined from three independent experiments (*n* = 3) for freeze-dried ethanol extracts prepared at a concentration ratio of 1:5 (corresponding to 0.2 g/mL, w/v). SD is standard deviation. Herbal extracts were compared with antibiotic controls. EPB, ethanol extract of *Piper betle* leaf; EBS, ethanol extract of *Bauhinia scandens* L. stem; ECO, ethanol extract of *Chromolaena odorata* leaf; OG, octyl gallate. Concentrations expressed in g/mL indicate the weight-to-volume (w/v) ratio of crude extract relative to ethanol during preparation.

The MICs of the herbal extracts against the reference strains are presented in Fig. 3. Among the extracts tested, EPB exhibited the strongest inhibitory activity against *S. aureus* (DMST 8840), showing the lowest MIC value (1,582.03 µg/mL). EBS displayed slightly lower activity (2,057.29 µg/mL), while OG demonstrated moderate inhibition (173.61 ± 60.14 µg/mL). A similar pattern was observed with *E. coli* (DMST 4212), where EBS (4,114.58 µg/mL) showed comparatively higher activity than EPB (12,656.25 µg/mL). OG and bacitracin were not effective against *E. coli* at the concentrations tested. The antibiotic controls, including enrofloxacin and cefazolin, yielded the lowest MIC values and served as positive benchmarks for assay validation.

### Minimum bactericidal concentration (MBC)

**Figure 4:**
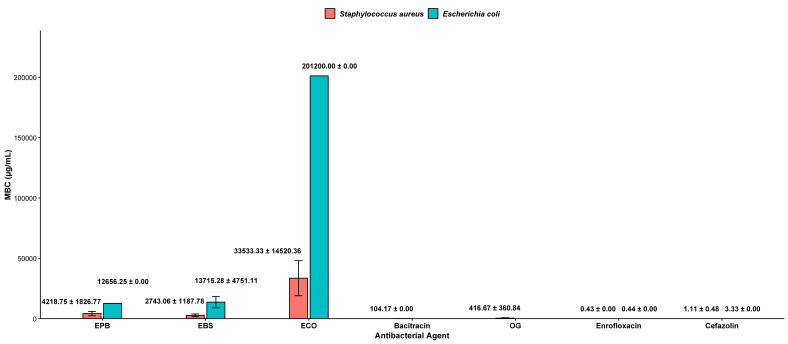
Minimum bactericidal concentration (MBC) test using PBL, BSL and CO for *S. aureus* and *E. coli*. Bars represent mean MBC values (µg/mL ± standard deviation [SD]) obtained from three independent experiments (*n* = 3) using freeze-dried ethanol extracts prepared at a concentration ratio of 1:5 (corresponding to 0.2 g/mL, w/v). Herbal extracts were evaluated alongside antibiotic controls. EPB, ethanol extract of *Piper betle* leaf; EBS, ethanol extract of *Bauhinia scandens* L. stem; ECO, ethanol extract of *Chromolaena odorata* leaf; OG, octyl gallate. Concentrations expressed in g/mL indicate the weight-to-volume (w/v) ratio of crude extract relative to ethanol during preparation.

The bactericidal activity of the herbal extracts is presented in Fig. 4. For *S. aureus* (DMST 8840), EBS showed the greatest efficacy among the herbal treatments, with an MBC of 2,743.06 ± 1,187.78 µg/mL. EPB also demonstrated bactericidal activity, though at a slightly higher concentration (4,218.75 ± 1,826.77 µg/mL). In comparison, OG exhibited a broader response range, with an MBC of 416.67 ± 360.84 µg/mL, reflecting variability in its bactericidal performance.

For *E. coli* (DMST 4212), both PBL and BSL required substantially higher concentrations to achieve complete bacterial killing, measuring 12,656.25 µg/mL and 13,715.28 ± 4,751.11 µg/mL, respectively. This pattern is consistent with the reduced susceptibility commonly observed in Gram-negative organisms. MBC values for OG and bacitracin were not determined for this strain. The antibiotic controls, including enrofloxacin and cefazolin, showed the lowest MBC values and served as positive assay standards.

### Bactericidal *versus* bacteriostatic effects of herbal extracts

As said in previous section, the MIC and the MBC for the herbal extracts are shown in Figs. 3 and 4, respectively. The MBC/MIC ratio was used to determine whether each extract exhibited bactericidal (MBC/MIC ≤ 4) or bacteriostatic activity. Ratios were calculated for *S. aureus* and *E. coli*, for which both MIC and MBC values were available.

All three herbal extracts demonstrated bactericidal activity against *S. aureus* (DMST 8840), as indicated by their MBC/MIC ratios, that can be found in Figs. 3 and 4. PBL showed a ratio of 2.67, BSL exhibited the lowest ratio at 1.33, and ECO presented a ratio of 1.00. Despite meeting the bactericidal threshold, ECO required substantially higher MIC and MBC values, indicating weaker overall potency.

For *E. coli* (DMST 4212), all extracts similarly displayed bactericidal MBC/MIC ratios, with values of 1.00 for PBL, 3.33 for BSL, and 2.00 for CO. However, the MIC and MBC concentrations required to inhibit and kill *E. coli* were substantially higher than those for *S. aureus*, indicating that the extracts were less effective against this Gram-negative bacterium.

### Correlation between herbal extract potency and antibacterial activity against *S. aureus* and *E. coli*

**Figure 5:**
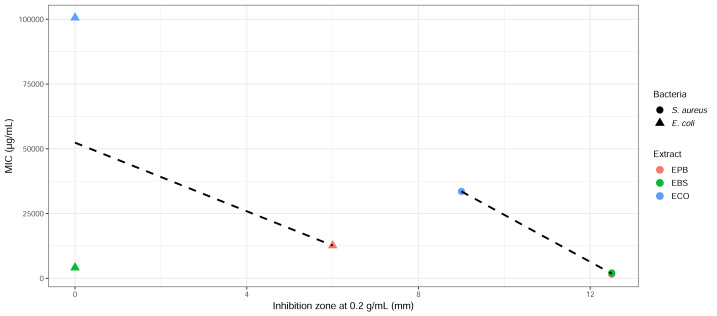
Correlation between inhibition zone diameter and MIC values for herbal extracts against *S. aureus* and *E. coli*. Scatterplot showing the relationship between inhibition zone diameters at 0.2 g/mL of the test- selected concentration and corresponding MIC values for the three herbal extracts (EPB, EBS, ECO) against *S. aureus* and *E. coli*. Each point represents one extract–bacterium combination, with colors indicating extract identity and shapes denoting bacterial species. A linear regression trendline (dashed) illustrates the overall association. Extracts with larger inhibition zones generally corresponded to lower MIC values, while those with no measurable inhibition (0 mm) showed substantially higher MICs, particularly for *E. coli*.

A correlation analysis was performed to compare inhibition zone diameters at 0.2 g/mL with the corresponding MIC values for each extract–bacterium pair as shown in Fig. 5. A negative relationship was observed, extracts that produced larger inhibition zones tended to exhibit lower MIC values, indicating a consistent relationship between disc-diffusion activity and broth microdilution performance. EPB and EBS showed moderate to strong inhibition of *S. aureus*, which aligned with relatively low MIC values (1,582.03–2,057.29 µg/mL), reflecting methodological differences between diffusion-based and broth-based assays and intrinsic bacterial resistance mechanisms. In contrast, ECO produced the smallest inhibition zones and had the highest MIC values. For *E. coli*, only EPB generated measurable inhibition, while EBS and ECO exhibited no zone formation and correspondingly high MIC values (>4,100 µg/mL). The overall correlation between inhibition zone and MIC was negative, supporting the expected trend that weaker disc-diffusion activity is associated with diminished inhibitory potency in broth microdilution assays.

## Discussion

The present study investigated the antibacterial activities of three ethanol-extracted herbal samples, named EPB, EBS, ECO, against *S. aureus* and *E. coli*, two of the most common mastitis-causing pathogens in dairy cattle. Overall, EPB and EBS demonstrated stronger inhibition, lower MIC values, and more favorable MIC–MBC relationships than ECO, aligning with earlier reports indicating that these plants contain high concentrations of phenolics and flavonoids with broad antimicrobial potential (Hossain et al., 2017; Indrianingsih et al., 2025; Singh et al., 2023). However, the absolute MIC and MBC values observed in this study were relatively high, which is an important consideration when evaluating their biological and practical significance.

The inhibition zone patterns at 0.2 g/mL supported these finds, with EPB and EBS formed larger zones against *S. aureus* compared with ECO, while all extracts showed weak to no activity against *E. coli*, indicating a higher natural resistance of Gram-negative bacteria (Uddin et al., 2021). The need for milligram-per-milliliter concentrations to achieve inhibition is not unexpected for crude plant extracts, as bioactive compounds constitute only a fraction of the total extract mass. Therefore, these MIC/MBC values should be interpreted as indicators of intrinsic antimicrobial potential rather than direct clinical applicability.

We selected the concentration of 0.2 g/mL for MIC and MBC testing because it produced clearer and more consistent inhibition than 0.1 g/mL in the disc diffusion assay. At 0.1 g/mL, inhibition of *S. aureus* showed greater variability, and inhibition of *E. coli* was inconsistent, with EPB showing a large SD and EBS/ECO producing no measurable zones. In contrast, at 0.2 g/mL all extracts generated reproducible inhibition against *S. aureus*, and EPB exhibited measurable and more consistent activity against *E. coli*. These results indicate that 0.2 g/mL represents the concentration at which all extracts demonstrated baseline antibacterial activity suitable for quantitative testing. Ngamsurach & Praipipat (2022) also reported in their study that the higher concentration of the herbal extracts produces more efficacy than the lower ones, as 400 mg/mL has better result than 300 mg/mL, 200 mg/mL and 100 mg/mL, respectively. In addition, selecting the stronger concentration aligns with recommended practice, where extract levels that produce measurable inhibition in diffusion assays are prioritized to avoid false negatives during broth microdilution (Saar et al., 2025), and we tested more than one for inhibition zone detection to seek for the adjustment before proceeding with the MIC test, which is currently mentioned in a site of the Clinical and Laboratory Standards Institute (CLSI, 2025).

The strong positive correlation observed between MIC and MBC values suggests that the bactericidal action of these extracts closely follows their inhibitory potential, which is consistent with the behavior of plant-derived antimicrobial compounds such as hydroxychavicol, quercetin derivatives, and terpenoids (Singh et al., 2023). EPB showed the most efficient performance, requiring comparatively lower concentrations to inhibit and kill *S. aureus*, which supports existing evidence describing *P. betle* L. as a potent antimicrobial herb widely used in traditional medicine (Ngamsurach & Praipipat, 2022). For EBS, the MIC and MBC values were moderately higher but still within effective ranges reported in other Bauhinia species (Islam et al., 2022). We observed it is likely that ECO consistently showed the weakest activity, likely due to variation in active compound abundance depending on plant origin, extraction method, or environmental factors. However, *C. odorata* has been widely documented to exhibit diverse biological activities, including antidiabetic, anticancer, anti-inflammatory, antimicrobial, antiparasitic, analgesic, antipyretic, and wound-healing effects (Olawale, Olofinsan & Iwaloye, 2022).

The inverse relationship observed between inhibition zone diameter and MIC reaffirmed that extracts with larger inhibition has required lower concentrations to suppress bacterial growth. This type of inverse relationship is commonly reported in phytochemical susceptibility tests and supports the reliability of diffusion-based screening before MIC determination (CLSI, 2024; Khoshkhounejad et al., 2021).

From a practical perspective, the high MIC/MBC value indicate that these crude extracts are unlikely to serve as stand-alone antimicrobial treatments, particularly against *E. coli*, where Gram-negative outer membrane barriers and efflux systems limit extract uptake (Zgurskaya & Rybenkov, 2020). Instead, EPB and EBS represent promising source materials for further optimization, including phytochemical fractionation, synergistic testing with antibiotics and *in vivo* evaluation. These steps are essential before considering application in mastitis control strategies.

## Conclusions

Our study demonstrates that the three herbal extracts showed distinct antibacterial patterns, with the strongest and most consistent activity directed against *S. aureus*. Among them, the *P. betel* L. extract exhibited the highest overall efficacy, followed by the *B. scandens* extract, while the *C. odorata* extract showed weaker and more variable effects. The extracts were far less active against *E. coli*, which aligns with the well-known resistance mechanisms of Gram-negative bacteria.

The agreement between disc diffusion results, MIC and MBC data, and the observed inverse relationship between inhibition zone size and MIC values provides strong support for the reliability of the antibacterial trends identified in this work. Collectively, these findings suggest that the extracts, particularly those derived from Piper betel and *B. scandens*, hold promise as potential plant-based alternatives or complementary agents for managing infections caused by *S. aureus*. Their limited activity against *E. coli*, however, indicates that they are unlikely to function as broad-spectrum treatments. Further studies should include bioassay-guided phytochemical fractionation to identify active compounds and improve antibacterial potency, particularly given the relatively high MIC and MBC values observed for the crude extracts. In addition, synergistic testing with conventional antibiotics is warranted, especially against Gram-negative pathogens where limited activity was detected. Evaluation against clinical and antimicrobial-resistant field isolates, followed by *in vivo* validation in mastitis models to assess safety, pharmacokinetics, and therapeutic efficacy, will be essential before considering practical application in dairy mastitis control.

##  Supplemental Information

10.7717/peerj.21134/supp-1 Supplemental Information 1 Raw Data and Pre-analysis data (Inhibition Zone)

10.7717/peerj.21134/supp-2 Supplemental Information 2 Raw Data and Pre-analysis data (MIC and MBC) in ug per mL

## Additional Information and Declarations

### Data Availability

The following information was supplied regarding data availability: The raw data and measurements are available in the Supplemental Files.
